# Ecological Adaptations of Gut Microbiota Members and Their Consequences for Use as a New Generation of Probiotics

**DOI:** 10.3390/ijms22115471

**Published:** 2021-05-22

**Authors:** Tereza Kubasova, Zuzana Seidlerova, Ivan Rychlik

**Affiliations:** Veterinary Research Institute, Hudcova 70, 621 00 Brno, Czech Republic; kubasova@vri.cz (T.K.); seidlerova@vri.cz (Z.S.)

**Keywords:** gut, microbiota, chicken, human, pig, probiotics

## Abstract

In this review, we link ecological adaptations of different gut microbiota members with their potential for use as a new generation of probiotics. Gut microbiota members differ in their adaptations to survival in aerobic environments. Interestingly, there is an inverse relationship between aerobic survival and abundance or potential for prolonged colonization of the intestinal tract. Facultative anaerobes, aerotolerant *Lactobacilli* and endospore-forming Firmicutes exhibit high fluctuation, and if such bacteria are to be used as probiotics, they must be continuously administered to mimic their permanent supply from the environment. On the other hand, species not expressing any form of aerobic resistance, such as those from phylum Bacteroidetes, commonly represent host-adapted microbiota members characterized by vertical transmission from mothers to offspring, capable of long-term colonization following a single dose administration. To achieve maximal probiotic efficacy, the mode of their administration should thus reflect their natural ecology.

## 1. Introduction

Gut microbiota of each warm-blooded omnivorous animal, both avian and mammal, consists of approximately 1000 different bacterial species. Taxonomically, these species belong to two major phyla, Firmicutes and Bacteroidetes, followed by two minority phyla, Proteobacteria and Actinobacteria, followed by phyla representatives that can be found only in some individuals (Verrucomicrobia, Spirochetes, Fusobacteria, Elusimicrobia or Synergistetes) [1,2,3,4]. Each bacterial species present in gut microbiota has been subjected, as a metaorganism together with its host, to natural selection over millions of years of evolution. If the bacterial species were to negatively affect host performance, the host would not reach sexual maturity or would produce lower numbers of offspring, resulting in fewer microbiota capable of being passed on. The host species has therefore repeatedly selected against any negative microbiota and selected for the core microbiota which we record at present.

The core microbiota consists of bacteria beneficial for its host. When individual microbiota members are obtained in pure cultures and provided back to its host to improve its performance, such cultures are called as probiotics. By definition, probiotics are live microorganisms that, when administered in adequate amounts, confer a health benefit on the host. Their benefits are expressed at multiple levels. Probiotics may degrade feed components difficult for their host to digest into easily metabolized organic acids [5,6]. Release of organic acids also decreases pH, which can suppress the expression of virulence factors of pathogens such as *Salmonella enterica* [7,8]. Probiotics may affect the rest of gut microbiota by production of antimicrobial substances [9]. Additional metabolic byproducts of probiotic strains, i.e., in addition to the dominant organic acids, may act positively on human or animal performance [10]. Probiotics can also modulate the immune response of their host [11] or positively affect gut physiology associated with more efficient nutrient resorption [12,13].

The most frequently used probiotics nowadays belong mainly to genera *Lactobacillus* and *Bifidobacterium* [14]. These genera are characterized by the production of organic acids that decrease pH and thus suppress the growth of competing microbiota [15]. *Lactobacilli* are common in milk-fermented products, which are beneficial for animal or human hosts [16,17]. *Lactobacilli* also commonly produce antimicrobial peptides, which may inhibit the growth of competing microbiota [18,19]. Different *Lactobacillus* species and their metabolic products exhibit immunomodulatory activities on vertebrate hosts [20,21], and supplementation of *Bifidobacterium* and *Enterococcus* probiotics decreased allergic rhinitis symptoms in children [22]. Positive effects of *Lactobacilli* and *Bifidobacteria* on human or animal gut health have been therefore repeatedly shown.

**Figure 1:**
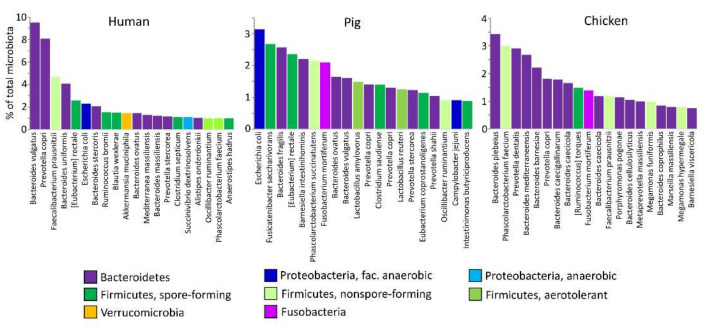
The twenty most abundant bacterial species present in gut microbiota of humans, pigs and chickens. Microbiota composition was determined in 44 human, 50 pig and 37 chicken samples processed in the authors’ laboratory within the last two years, and the most abundant operational taxonomic units (OTUs) were identified. Two *Lactobacillus* species were common in pigs, while in humans and chickens, not a single *Lactobacillus* species was present among the twenty most abundant species. Neither *Bifidobacterium* nor any other Actinobacteria representative was present among the top 20 OTUs in any of these hosts.

However, gut microbiota consists of hundreds of bacterial species with a neutral to positive relationship with its host, and there is no justification why additional species from other genera could not be used as well. Data from microbiota studies using next generation sequencing show that *Lactobacilli* dominate in the small intestine, but in the caecum or colon, *Lactobacilli* and *Bifidobacteria* do not represent the dominant microbiota members [3,23,24,25]. Instead, other genera and species are more characteristic for distal parts of the intestinal tract, and this is common among distantly related species such as humans, pigs or chickens (Figure 1). Despite this, *Lactobacilli* and *Bifidobacteria* are used in all these species as probiotics [26,27,28],. In some cases, even the same species, such as *Lactobacillus plantarum*, has been tested in different hosts [29,30,31]. However, at least in chickens, orally administered *Lactobacilli* do not permanently colonize the intestinal tract [32]. It can be argued that humans already have had a positive experience with *Lactobacilli* or *Bifidobacteria* as probiotics [33]. However, considering candidate probiotic gut microbiota without any bias, prior human experience with currently used probiotics cannot compare to evolution and natural selection, which have been operating for millions of years. This why in this review we remind readers that corrective actions towards intestinal disorders may also be achieved with other gut microbiota members; we carefully propose that if the function and ecology of the other microbiota members is understood and followed, benefits for the animal hosts might be even higher than after administration of lactic acid probiotics.

## 2. Novel Types of Probiotics from Gut Microbiota

**Figure 2:**
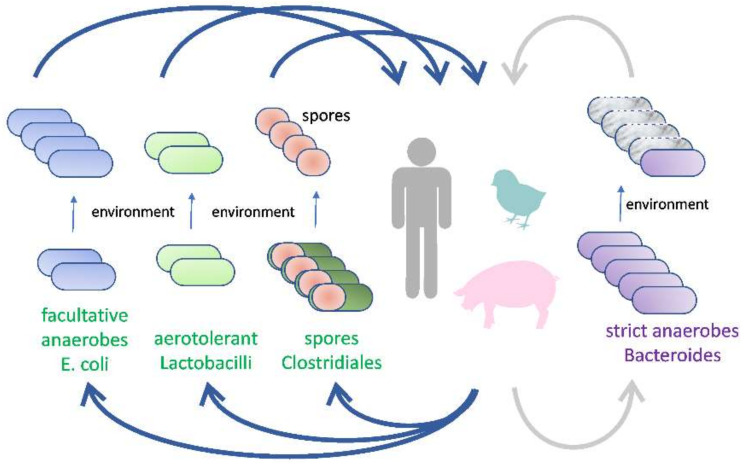
Gut microbiota and their adaptation to air survival. There are different forms of adaptation to air exposure. Aerotolerant bacteria or facultative anaerobes survive aerobic exposure in the form of vegetative cells. Another group of gut microbiota survives air exposure in the form of spores. The last group of gut anaerobes, mostly from Bacteroidetes, did not evolve any specific form of air survival and quickly loses viability after air exposure, which negatively affects their transmission via the environment.

If novel types of viable probiotic strains are used for the improvement of gut function, they should originate from gut microbiota, i.e., there should be evidence that these can colonize the intestinal tract. Moreover, to increase the likelihood of their safety, these bacteria should belong to the core microbiota of the given host. Although one cannot exclude that even non-intestinal bacteria may trigger responses in the intestinal tract following their ingestion, e.g., as in the case of intoxication by *Bacilli* contaminated food [34], such interactions are specific and beyond the scope of this review. Instead, we focus on bacterial species that are common to the intestinal tract, as these species have adopted strategies to survive and multiply in the gut, and consequently, they interact well with their host and affect its performance. We do not focus on the importance of their taxonomic classification and instead stress their biology and ecology in selecting novel candidate probiotics. Using this approach, there are two basic groups of gut microbiota: (a) those capable of long-term survival after air exposure and (b) genuine gut anaerobes not expressing any specific form of aerobic resistance. Since there are different forms of adaptation to air exposure, four different groups of gut microbiota can be defined: (1) aerotolerant bacteria commonly associated with food and feed, (2) facultative anaerobes, (3) spore-forming gut anaerobes and (4) non-spore-forming gut anaerobes (Table 1, Figure 2).

### 2.1. Aerotolerant Bacteria Commonly Associated with Food and Feed

This group comprises mainly lactic acid bacteria and genera *Lactobacillus* and *Bifidobacterium*, in particular. Lactic acid bacteria are the most frequently used probiotics nowadays. *Lactobacilli* are low GC content bacteria (around 36% GC content) with a genome of around 2 Mbp in size [35]. Due to their small genome size, *Lactobacilli* do not encode an extensive set of genes with broad functions, and instead they are specialists in the glycolysis of common saccharides and the release of organic acids, and consequently, they decrease the pH of their environment [15]. The pH in a *Lactobacilli*-fermented environment commonly decreases to values around 4.5, which prevents the growth of other microbiota. The more fermentable carbohydrates that are present in their environment, the faster the decrease of environmental pH [15]. *Lactobacilli* have been used as probiotics for centuries; however, sequencing data show that although *Lactobacilli* belong among common gut microbiota members in the small intestine, *Lactobacilli* do not dominate in the microbiota of distal parts of the digestive tract [3,25,38,39,40]. Dominance of *Lactobacilli* in the small intestine, sometimes around 90% of all duodenal and jejunal microbiota, is due to their acid resistance, aerotolerance and rapid multiplication using energy from carbohydrate fermentation. There are different explanations for the probiotic activities of *Lactobacilli* [10]; however, there are also reports summarizing that two-thirds of the human population do not respond to *Lactobacilli* administration [41]. An explanation of the inconsistencies in the *Lactobacilli* probiotic effect is likely as follows. *Lactobacilli* in pure bacterial culture do not efficiently colonize the intestinal tract [32]. *Lactobacilli* do not represent typical gut-adapted bacteria since *Lactobacilli* are aerotolerant, and aerotolerance is not necessary for gut-adapted bacteria due to the anaerobic conditions of the gut. *Lactobacilli* are primarily food or feed bacteria, highly preferring mammalian milk or plant carbohydrates for their metabolism [5,6,14,42]. *Lactobacilli* prefer microaerobic conditions and are therefore common in the vaginal microbiota of humans or crop microbiota of chickens [36,38,43]. *Lactobacilli* are ubiquitously present in the external environment, e.g., in chicken bedding [44], and vertebrates are continuously exposed to and supplied with *Lactobacilli*. Their universal presence in the environment also means that their natural sources for vertebrates are high enough and *Lactobacilli* do not need to be supplied as probiotics for oral administration, except for very unusual cases of dysbiosis. Warm-blooded animals are adapted to a continuous supply of *Lactobacilli* and did not evolve mechanisms for long-term *Lactobacilli* colonization. *Lactobacilli* do not efficiently colonize the intestinal tract, and to be present, they have to be continuously supplied [10,45]. If this condition is not met, *Lactobacilli* will fail as probiotics.

Why then are *Lactobacilli* generally accepted as safe and of beneficial effect? *Lactobacilli* may degrade oligosaccharides that are difficult for their host to digest into easily metabolized organic acids [5,6]. *Lactobacilli* may also digest other substrates such as gluten and gliadin [46]. Metabolic byproducts of *Lactobacilli*, i.e., in addition to the dominant lactic acid or other organic acids, may act positively on human or animal performance [10]. However, the most relevant probiotic property of *Lactobacilli* is the rapid decrease in pH. A pH below 5 inactivates the majority of competing bacteria and makes such an environment microbiologically safe for the human, pig or chicken host, containing non-pathogenic lactic acid bacteria only [15,33]. Since such pH cannot be achieved in distal parts of intestinal tract, the probiotic activity of *Lactobacilli* and *Bifidobacteria* (see below) is expressed primarily outside the host. *Lactobacilli*-fermented feed or food is microbiologically safe, which is central even nowadays in areas with lower hygienic standards and was even more important in the history of the human population 200 or more years ago when humans did not know anything about *Lactobacilli* but knew that fermented food was safe and therefore healthy [33]. Food- or feed-origin *Lactobacilli* [47] also means that *Lactobacilli* strains need not be host-specific and that *Lactobacilli* obtained from human feces can be used in pigs or chickens and vice versa—because *Lactobacilli* in all these hosts originate from carbohydrate rich food or feed.

*Bifidobacterium* is another genus known as a probiotic [5,6]. *Bifidobacteria* are phylogenetically distant from *Lactobacilli*. They have a small genome size like *Lactobacilli*, but unlike *Lactobacilli*, the genome of *Bifidobacteria* is characterized by a high GC content of around 63% [35]. *Bifidobacteria* exhibit a high level of resistance to different stress factors [48], which allows them to survive in an aerobic environment [35]. The principles of such resistance are not fully understood, although in some Actinobacteria, spore formation similar to that in Clostridia has been described [49]. Similar to *Lactobacilli*, *Bifidobacteria* also prefer carbohydrate catabolism [50] followed by a decrease of pH in their environment, thus suppressing multiplication of competitive microbiota. Unlike *Lactobacilli*, *Bifidobacterium* can colonize the chicken caecum after a single dose administration [32]. However, the abundance of *Bifidobacterium* is around 0.5% of total microbiota, i.e., much less than *Bacteroides* sp., which can form 10% of total microbiota using the same mode of administration [32].

### 2.2. Facultative Anaerobes

Another group of gut microbiota is represented by facultative anaerobes, and *E. coli* in particular is ubiquitously present, both in the environment and in the intestinal tract of nearly all warm-blooded animals. With a chromosome around 5 Mbp in size, *E. coli* belongs among gut microbiota with larger genomes, which enables it to encode and express multiple metabolic pathways [2,3,25,38,40,51]. As a bacterium capable of aerobic growth, any local increase in oxygen species during host inflammatory response does not affect *E. coli* to as much of an extent as vegetative cells of strict anaerobes. *E. coli* therefore increase during dysbiosis [38,52], although this may be both the cause as well as the consequence of an increase of oxygen species. *E. coli* also tend to overgrow in liquid anaerobic cultures inoculated with fecal material [15]. Unlike *Lactobacilli*, this is not caused by a decrease in pH but rather by its broad metabolic capacity, including anaerobic respiration, short generation time and use of nutrients, which become limited for other microbiota. Since *E. coli* is commonly available in the external environment, its use as a probiotic is questionable. However, in the youngest individuals during the first hours of life, administration of commensal *E. coli* may act in preventing colonization with pathogenic clones. This is the reason for the use of *E. coli* Nissle in human infants [53,54]. Administration of commensal *E. coli* might also be considered as a part of activities towards the decrease of antibiotic resistance in microbial populations. *E. coli* serves as a reservoir of many horizontally transmissible genes responsible for antibiotic resistance for different Gram-negative pathogens, such as *Salmonella* [55]. Colonization of newborn individuals with antibiotic-sensitive *E. coli* may therefore interfere and reduce their colonization with antibiotic resistant clones, thus decreasing the spread of antibiotic resistance in microbial communities.

### 2.3. Spore-Forming Firmicutes

Spore-forming Firmicutes include Bacilli, Erysipelotrichia and Clostridia. Of these, Bacilli and genus *Bacillus* are only occasionally recorded in gut microbiota [34]. Despite this, spores of *Bacilli* have been tested in poultry or piglets as probiotics [56,57,58]. Since endospores are highly specific to prokaryotes, it is possible that structures present on their surface or associated with spore germination may activate the innate immune system and increase host resistance to enteric infections. On the other hand, the observed positive effects of *Bacillus* spore administration likely do not have anything to do with *Bacillus* multiplication and permanent colonization of the intestinal tract. Since *Bacilli* are common in the external environment [34,44,59], vertebrate hosts must have adapted to permanent contact with *Bacillus* spores, and if these are to be used as probiotics, they should be continuously administered to mimic the natural exposure of vertebrates.

Bacterial species from the remaining two classes, Erysipelotrichia and Clostridia, represent common gut microbiota members that belong to the core microbiota of many warm-blooded hosts. Though common in different hosts, recent findings point towards a specific mode of colonization due to their distinct life cycle associated with spore-formation. Spore-forming bacteria are rarely shared between genetically related individuals from different households, and instead, higher similarities in the composition of spore-formers are observed among genetically unrelated humans sharing the same household [60]. However, when metagenomic sequences were compared, those belonging to endospore-forming Firmicutes differed even between mothers and their children living within the same household [61,62], indicating independent colonization from environmental sources. Spore-forming Clostridiales exhibit increased variability yet tend to be less dominant members of the community [48,63,64] (Figure 1). In chickens, it is impossible to efficiently colonize newly hatched chicks with pure cultures of different endospore-forming Firmicutes [32], and the presence of Lachnospiraceae and Ruminococcaceae in the caecum of chicks from commercial hatcheries [2,24], i.e., in the birds without any contact with adult hens, is in fact direct evidence of an exclusive environmental origin of these bacteria. Spore formers are found mainly in these two families, although the inactivation of vegetative cells and the culture of spore-forming bacteria shows that family Lachnospiraceae represents a major spore-forming population in the human gut [37]. These bacteria are common to the intestinal tract and include major butyrate producers [35], yet it seems rather difficult to achieve successful colonization of the target host after experimental administration. An explanation of all of these observations is that due to prolonged survival of endospores in the environment - these bacteria do not need to colonize the intestinal tract permanently after every possible opportunity. Instead, these species are continuously supplied from the environment, and a balance between environmental supply, anaerobic replication in the gut and excretion back to the environment allows them to persist in the gut. Due to their ecology and environmental origin, demands on their origin need not to be that strict, and human isolates might be of similar efficacy if used in pigs or chickens. However, if these species are to be used as probiotics, they will have to be continuously supplied. Despite this, it will have to be kept in mind that experimental supplementation will always compete with a continuous supply of endospore-forming Firmicutes from the environment, which may compromise and interfere with the probiotic effect after experimental intervention. Since the natural spread of this type of bacteria is via endospores, their supplementation in the form of endospores rather than in the form of vegetative cells should be considered. Once their supplementation is finished, they may gradually disappear from the intestinal tract. Endospore-forming Firmicutes represent a specific group of gut anaerobes. Interestingly, there are Lachnospiraceae or Ruminococcaceae members which lost the ability to form endospores, e.g., *Faecalibacterium*, *Oscillibacter* or *Roseburia* [65]. It will be interesting to follow the ecology and natural dissemination of these genera to learn more about novel types of adaptation evolving after the loss of sporulation capacity.

### 2.4. Non-Spore-Forming Gut Anaerobes

Non-spore-forming gut anaerobes belong mainly to phylum Bacteroidetes but can also be found among Proteobacteria, Verrucomicrobia, Fusobacteria, Spirochaetes, Elusimicrobia or Synergistetes. There is also an order within Firmicutes, Selenomonadales, isolates of which lost the ability of spore-formation, and instead, captured genes which enable them to produce an outer membrane characteristic of Gram-negative bacteria [66]. All these bacteria do not express any specific form of adaptation to an aerobic environment, which influences their ecology. Non-spore-forming gut anaerobes are less likely to be found across multiple individuals than those capable of spore formation [48], and in agreement, host-dependent adaption of different species within genus *Bacteroides* has been described [67]. Bacteria no longer capable of sporulation are usually less prevalent but more abundant compared to spore-formers, suggesting an increase in colonization capacity [68]. *Bacteroides* species are vertically transferred from mothers to offspring, both in humans and chickens [24,61]. These bacteria are efficiently transferred by fecal transplantation in humans [69], and if administered to newly hatched chicks, they efficiently colonize the caecum after a single dose administration [32,70]. Interestingly, despite host adaptation, chicks can be equally colonized by both chicken- and human-adapted species [32], although only the chicken-adapted species will remain present until adulthood [67]. Their inability to extend environmental survival is therefore compensated for by their efficient colonization at the first opportunity and the slightly higher oxygen resistance of their vegetative cells than vegetative cells of spore-forming Clostridiales [35]. The slightly increased survival of vegetative cells is, however, incomparable with spore resistance. Since these bacteria are usually present at higher abundance in gut microbiota, they are also excreted in the feces in higher abundance, which increases the probability of reaching a new host. If the vertical transfer these microbiota members is affected, such as in caesarean section-delivered infants [71,72] or in chicks in commercial production hatched in the absence of adult hens [24], environmental sources are usually not rich enough to provide such bacteria. These are the reasons why these bacteria should be considered as the targets for novel types of probiotics. Due to their evolutionary adaptations, a single dose of administration is usually enough to restore their presence in the digestive tract. All of this suggests that the non-spore-forming strict anaerobes from genera *Bacteroides*, *Prevotella*, *Parabacteroides*, *Barnesiella*, and *Alistipes*, but also those from *Sutterella*, *Parasutterella*, *Succinatimonas*, *Akkermansia*, *Phascolarctobacterium*, *Megamonas*, *Megasphaera*, *Veillonella* or *Dialister*, are promising candidates for novel types of probiotics.

## 3. Final Remarks

**Figure 3:**
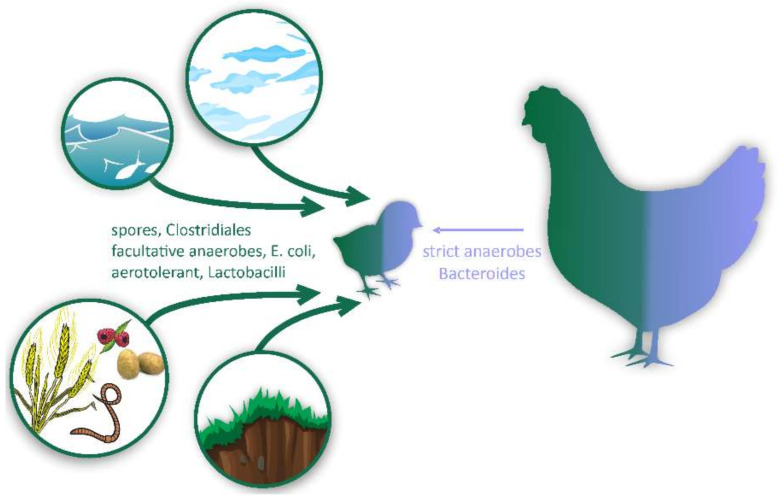
Sources of gut microbiota for chickens. Despite continuous supply of aeroresistant gut colonizers from the environment, these usually represent around 50% of microbiota in chickens (or humans or pigs). On the other hand, strict anaerobes without any adaptation to air survival are transferred by less frequent contact, and despite this, usually represent the second half of gut microbiota. If any of the gut microbiota is considered as probiotic, natural ecology and adaptations should be considered and followed.

There is an inverse correlation between the environmental survival of microbiota species and their ability to colonize their host (Table 2, Figure 3). Microbiota members capable of prolonged environmental survival do not efficiently colonize the intestinal tract, and if used as probiotics, they must be provided continuously to reflect their natural ecology. Administration of pure cultures of *Lactobacilli* are of minimal effect on host performance, although we admit that during continuous supply even the non-colonizing bacteria may trigger a host response. *Lactobacilli* and *Bifidobacteria* act on a different level, at food and feed fermentation. In all cases, the fate of strains used as probiotic supplements should be exactly followed. Mere bacterial plating of *Lactobacilli* is not sufficient because each individual is positive for these bacteria from environmental sources. Instead, isolate-specific PCR is highly recommended.

The use of additional bacterial species belonging to core gut microbiota of a particular host as novel types of probiotics should be as safe as the use of *Lactobacilli* and *Bifidobacteria*. Core microbiota members were selected during evolution over millions of years, i.e., for much longer than human experience with lactic acid bacteria [33]. This fact supports, favors and justifies the use of a broader spectrum of gut microbiota species as probiotics. Nevertheless, a precautionary principle must be followed, and each strain to be used as probiotics must be tested individually and sequenced completely since commensals such as *E. coli* may become a serious pathogen following acquisition of only a few genes by horizontal gene transfer. Alternatively, some bacterial species may behave as commensal in one host but as a pathogen in another host. Since spore-forming and aerotolerant gut microbiota members are ubiquitously present in the environment in high quantities, in addition to some specific cases, they are less likely to be effectively used as probiotics with a reproducible effect. On the other hand, gut microbiota members with poor environmental survival, high host adaptation and potential to permanently colonize after a single dose of administration should be considered as novel types of probiotics. These can be used to stimulate microbiota development in young animals or to shorten the restoration period and recovery of normal gut microbiota after episodes of dysbiosis.

## 4. Conclusions

There are hundreds of bacterial species colonizing the intestinal tract, and nearly any of them can be tested as a novel type of probiotics. There is no reason to restrict probiotics to *Lactobacilli* or *Bifidobacteria*. However, when testing current or considering novel types of probiotics, understanding their biological functions is important for achieving maximal effect. Here we reminded how oxygen resistance of probiotic strains may influence their administration. Bacterial species resistant to oxygen usually have to be provided continuously since they poorly colonize the intestinal tract. On the other hand, species with no specific adaption to oxygen exposure usually colonize for a prolonged period of time after a single dose administration.

## Figures and Tables

**Table 1:** Functional classification of the most frequent gut microbiota members according to their adaptations to air exposure.

Form of Air Resistance	Ecological Classification	Major Taxa from Gut Microbiota	Reference
Aerotolerance	Food and feed microbiota	*Lactobacilli*, Actinobacteria	[14]
Facultative anaerobes	Ubiquitous distribution	Enterobacteriaceae, *E. coli*	[35,36]
Spore formation	Gut microbiota	Clostridiales	[32,35,37]
None	Gut microbiota	Bacteroidetes, Selenomonadales	[35]

**Table 2:** Summary of ecological adaptations of gut microbiota members and consequences for probiotic administration and host colonization.

Form of Air Resistance	Major Taxa	Vertical Transmission	Environ. Origin	Host Adaptation	Probiotic Dosage	Permanent Colonization
None	Bacteroidetes, Selenomonadales	Yes	No	Yes	Single	Yes
Aerotolerance	Lactobacilli, Actinobacteria	No	Yes	No	Repeated	No
Fac. anaerobes	Enterobacteriaceae	No	Yes	No	Repeated	No
Spores	Clostridiales	No	Yes	No	Repeated	No

## Abbreviations

OUT, operational taxonomic unit; WCHA, Wilkins—Chalgren agar; BHI, brain heart infusion.
